# Assessment of estimated and measured resting metabolic rates in type 1 and type 2 diabetes mellitus

**DOI:** 10.1016/j.heliyon.2024.e28248

**Published:** 2024-03-19

**Authors:** Akin Dayan, Nilay Ergen, Sami Sabri Bulgurlu

**Affiliations:** a Department of Family Medicine, Diabetes Polyclinic, Haydarpasa Numune Training and Research Hospital, Istanbul, Turkey; b Department of Physiology, Yeni Yuzyil University, Istanbul, Turkey; c Department of Family Medicine, Minister of Health, Family Health Medicine Center, Istanbul, Turkey

**Keywords:** Calorimetry, Indirect, Energy expenditure, Diabetes mellitus, Insulin-dependent

## Abstract

**Aim:**

This study aimed to compare the estimated and measured resting metabolic rates of patients with type 1 and type 2 diabetes mellitus in an outpatient clinical setting.

**Material and methods:**

Participants were categorized into three groups that included type 1 diabetes, type 2 diabetes, and individuals without diabetes. Bland–Altman analysis was used to identify the equation that most accurately predicted the measured resting metabolic rates. Multiple regression analysis was used to identify the factors affecting resting metabolic rates.

**Results:**

Resting metabolic rates was observed to be higher in subjects with type 2 diabetes compared to that of the other groups. There was a proportional bias between predicted and measured resting metabolic rates. Type 1 diabetes, type 2 diabetes, male sex, body weight, waist circumference, and triglyceride level were factors that positively predicted resting metabolic rates, and age was a factor that negatively predicted it.

**Conclusions:**

Although there was a bias between estimated and measured RMR, the most accurate results were achieved with the Mifflin–St Jeor equation for women with type 1 diabetes, with the Owen equation for men with type 1 diabetes, with the Harris Benedict equation for women with type 2 diabetes, and with the Ikeda equation for men with type 2 diabetes as well as for women and men in the control group.

## 1 Introduction

Regulation of glucose levels plays a major role in reducing early mortality and complications in diabetes. The first-line approach for the regulation of blood glucose levels involves medical nutrition therapy. Devising personalized medical nutrition therapy requires an assessment of metabolic rate.

The major components of daily energy expenditure include resting metabolic rate (RMR), diet-induced thermogenesis, and activity thermogenesis. RMR accounts for 60%–75% of total energy expenditure, and diet-induced thermogenesis accounts for 10%–15% [1]. Physical activity is the most variable component of energy expenditure and is responsible for up to 20% of energy expenditure in sedentary individuals, whereas its share is higher in active individuals [2].

RMR plays a major role in the regulation of body weight [3]. Indirect calorimetry (IC) is considered the gold standard for measuring resting energy expenditure based on oxygen consumption (VO_2_) and carbon dioxide production (VCO_2_) [4]. However, it is not possible to use IC for every patient in an outpatient clinical setting, and based on this several formulas have been developed to allow for practical and accurate measurement of RMR using age, body weight, height, and gender [5].

Compared to people without diabetes, patients with type 1 and type 2 diabetes have been reported to possess higher RMR. Increased RMR in diabetic patients is attributed to increased catabolic rate, hyperglucagonemia, and gluconeogenetic activity [3,[6], [7], [8]]. Insulin deficiency in people with type 1 diabetes mellitus (DM) leads to significant metabolic changes, including increased RMR and reduced mitochondrial function [9]. Increased sympathetic activity also contributes to high RMR [10].

There are previous studies in the literature that measure RMR and estimated RMR in healthy volunteers and type 2 diabetics. However, the number of studies conducted with type 2 DM is limited, and the research results are contradictory [[11], [12], [13]]. Research examining Type 1 DM and RMR is also scarce, particularly studies comparing estimated RMR [14,15]. There is a current knowledge gap regarding this subject in the literature.

In many studies, easily accessible and well-known equations such as Harris Benedict equation (HBE), Mifflin–St Jeor equation (MSJ) and Owen equation (OW) have been used [11,16,17]. More accurate results were obtained with MSJ in type 2 diabetics [18]. The Ikeda equation was also included in our study because a small number of type 1 DM were included in the study along with type 2 DM, and body mass index (BMI), one of the factors affecting RMR, was similar in our type 1 DM and control groups [19]. There is no study in the literature examining this subject that includes control, type 1, and type 2 DM groups and that possesses this sample size. Additionally, using equations including free fat mass (FFM) and fat mass (FM) in the outpatient clinic is impractical. There was no difference between the anthropometric (height and weight) and body composition (FM and FFM) models used to estimate energy consumption in the study with type 1 DM and control groups [20]. In our study, we attempted to examine the well-known equations that can be easily calculated such as age, gender, body weight, and height. This study aimed to investigate the correlation between measured RMR and estimated RMR based on practical predictive equations, such as HBE, MSJ, and OW and Ikeda, that can be used in outpatients with and without type 1 and type 2 DM and to identify factors that predict RMR.

## 2 Material and methods

### 2.1 Patients and sample size

This study is an analytical cross-sectional study. Patients were included in the study by simple random sampling from patients within five years who were referred from diabetes, family medicine and internal medicine policlinics to the physiology laboratory of Numune Training and Research Hospital, Istanbul, Turkey, regardless of the seasons. The five-year population of the physiology laboratory was 1500 patients, and the minimum sample size was calculated as 305 patients. The study enrolled 395 of 432 subjects randomly selected from among patients who met the inclusion criteria. The subjects were categorized into three groups, that included type 1 DM, type 2 DM, and individuals without DM, and they were also classified by gender.

Volunteers participating in the study are considered to be Caucasian residents. The study received approval from the Ethics Committee of Haydarpaşa Numune Training and Research Hospital (approval no. 2010/012). The study was conducted according to the Declaration of Helsinki, and written informed consent was obtained from the patients. Patients were informed of the procedures to be applied.

### 2.2 Inclusion and exclusion criteria

The study enrolled participants aged 18–80 with normal thyroid function tests and without chronic renal disease, general condition disorders, and dementia, both with and without type 1 and type 2 DM. The diagnosis of type 1 and type 2 DM was made according to the criteria of the American Diabetes Association [21].

Subjects were excluded if they were pregnant, engaged in professional sports, had thyroid dysfunction or used thyroid medications, had chronic liver disease, had stage C and D heart failure according to American Heart Association classification, used medications that may disrupt metabolic control (cortisol, immunosuppressive therapy, and others), had myocardial infarction during the last year, or had any infectious disease, malignancy, or acute or chronic respiratory disease. The patients continued their routine antidiabetics, antihypertensive, and antihyperlipidemic medications. The patients were invited to the clinic one day before without exercising, alcohol, and smoking. Women who were menstruating or would menstruate the next day were invited on subsequent days.

### 2.3 Physical examination and laboratory measurements

Subjects underwent body weight measurements while wearing light clothing and height measurements and waist circumference measurements using a standardized measuring tape. Their blood pressure and pulse rates (PR) were also measured after rest using a calibrated Erka brand device. Routine physical examinations of the patients were performed.

Many factors influence RMR, and independent variables were determined based on the aim of our study, evaluating other studies in the literature [14,17,22,23].

Subjects were further tested for fasting blood glucose (FBG), Hemoglobin A1c (HbA1c), creatinine, total cholesterol, high-density lipoprotein (HDL)-cholesterol, triglyceride, and low-density lipoprotein (LDL)-cholesterol levels using blood collected on the same day after 10–12 h of fasting. HbA1c levels were measured using ion-exchange high-performance liquid chromatography (Tosoh Bioscience, G7 Automated HPLC Analyzer).

### 2.4 Resting metabolic rate measurement

The dependent variable, participant RMR values, were measured according to Cosmed's FitMate Metabolic System (Rome, Italy) using IC (based on measurement of oxygen utilization). FitMate is a metabolic analyser that calculates energy expenditure based on oxygen consumption [24,25]. Subjects underwent measurements after 10–12 h of fasting and no exercise in the morning (08:00–10:00) in a rested state in a thermoneutral environment with appropriate humidity and pressureby breathing into a mask for 15 min in the supine position after 30 min of rest. The tests were conducted in the same physiology room with air-conditioned constant humidity and a temperature of 20–22 °C. (steady state).

### 2.5 Estimated RMR

Estimated RMR was determined using four equations, that are practical and easy to calculate in an outpatient clinical setting [5,[26], [27], [28]].

Harris and Benedict [26].

Female 655.1+(9.56 × weight)+(1.85 × height)-(4.68 × age),

Male 66.47+(13.75 × weight)+(5.0 × height)-(6.75 × age)

Mifflin et al. [5].

Female (10 x weight)+(6.25 x height)-(5 x age) −161.

Male (10 x weight)+(6.25 x height)-(5 x age)+5.

Owen [27,28].

Female (7.18 × weight)+795,

Male(10.2 × weight)+879.

Ikeda [19].

(10 × weight)-(3 × age)+(125 × sex)+750,

Weight: kilograms; height: centimeter; age: years; male:1; female:0.

### 2.6 Statistical analyses

Statistical analysis was performed using SPSS 18.0 for the Windows program. Categorical variables were presented using numbers and percentages, whereas numerical variables were presented using descriptive statistics (mean and standard deviation). Distribution analysis was performed according to skewness, kurtosis, histograms, and the Kolmogorov–Smirnov test. Intergroup categorical comparisons were performed using the Chi-square test, and numerical comparisons were performed using ANOVA for normally distributed samples Tukey's B test for pairwise analysisand KruskaWallis test for independent groups for which the assumption was not met. Pairwise comparisons were adjusted using Bonferroni correction and were subjected to the Mann–Whitney *U* test.

The difference between the RMR estimated using equations and the RMR as measured with IC was calculated (estimated RMR − measured RMR). The percentage of deviation between estimated RMR and measured RMR was calculated using the formula (estimated RMR – measured RMR)/(measured RMR) × 100, and the mean RMR was calculated using the formula (estimated RMR + measured RMR)/2. The *t*-test was used to determine if the difference between the estimated and measured RMR was statistically significant. When the significance level was p > 0.05, H_0_ was accepted and Bland–Altman plot analysis was performed with lower and upper limits of agreement. Analysis results were evaluated visually and using linear regression.

Multivariate linear regression analysis was used to identify factors predicting RMR. The independent variables were constructed taking into account other RMR studies and our population. As independent variables, the model included DM status (type 1 or type 2), gender, age, height, body weight, waist circumference, systolic blood pressure (SBP), diastolic blood pressure (DBP), PR, FBG, HbA1c, blood creatine, HDL cholesterol, and triglyceride parameters.

Statistical significance was set at p < 0.05.

## 3 Results

The study included 395 participants, comprising 26.8% (n: 106) of subjects with type 1 diabetes, 29.1% (n: 115) with type 2 diabetes, and 44.1% (n:174) controls.

Measured and estimated RMR values in the control and patient groups are provided in Table 1.

**Table 1:** RMR values measured and calculated in the control and diabetic groups.

Variables	Gender	Total	Control	Type 1 DM	Type 2 DM	P
Measured RMR (kcal/day)	All	1470.60 ± 324.28	1400.09 ± 283.03^a^	1467.33 ± 319.99^b^	1580.29 ± 357.53^a,b^	**<0.001**
	Female	1414.57 ± 290.25	1364.15 ± 260.47^a^	1346.73 ± 260.73^b^	1548.62 ± 316.67^a,b^	**<0.001**
	Male	1608.69 ± 361.76	1572.60 ± 325.79	1597.39 ± 329.18	1658.97 ± 438.86	0.615
RMR/Weight (kg)	All	20.30 ± 4.05	19.61 ± 3.35^a^	22.97 ± 4.57^a,c^	18.87 ± 3.33^c^	**<0.001**
	Female	19.91 ± 3.78	19.64 ± 3.48^a^	22.16 ± 4.28^a,c^	18.89 ± 3.34^c^	**<0.001**
	Male	21.24 ± 4.54	19.46 ± 2.65^a^	23.85 ± 4.76^a,c^	18.82 ± 3.35^c^	**<0.001**
HBE (kcal/day)	All	1524.13 ± 222.62	1499.71 ± 214.40	1526.07 ± 199.48	1559.30 ± 250.42	0.083
	Female	1445.39 ± 145.02	1446.31 ± 154.01^a^	1395.66 ± 107.01^a,c^	1477.13 ± 143.16^c^	**0.005**
	Male	1718.22 ± 259.35	1755.99 ± 274.16	1666.72 ± 179.75	1763.47 ± 333.53	0.161
MSJ (kcal/day)	All	1434.96 ± 224.21	1408.91 ± 220.74	1451.95 ± 209.11	1458.71 ± 240.10	0.119
	Female	1346.08 ± 163.41	1351.77 ± 174.42	1297.78 ± 126.30^b^	1368.47 ± 160.71^b^	**0.038**
	Male	1654.04 ± 202.58	1683.17 ± 215.95	1618.21 ± 142.34	1682.94 ± 259.40	0.238
OW (kcal/day)	All	1417.27 ± 208.31	1374.48 ± 196.32^a^	1397.15 ± 190.41^b^	1500.56 ± 218.91^a,b^	**<0.001**
	Female	1315.92 ± 115.61	1304.73 ± 115.12^a,b^	1237.42 ± 72.36^a,c^	1388.23 ± 98.05^b,c^	**<0.001**
	Male	1667.08 ± 172.28	1709.28 ± 156.41^a^	1569.40 ± 110.23^a,c^	1779.69 ± 183.57^c^	**<0.001**
İkeda Equation (kcal/day)	All	1401.22 ± 173.83	1380.34 ± 176.62^a^	1367.02 ± 143.46^b^	1464.33 ± 179.93^a,b^	**<0.001**
	Female	1349.65 ± 149.29	1342.95 ± 155.15^a,b^	1274.60 ± 102.14^a,c^	1411.77 ± 140.66^b,c^	**<0.001**
	Male	1528.32 ± 165.00	1559.80 ± 164.71^a^	1466.69 ± 111.46^a,c^	1594.94 ± 201.44^c^	**0.001**

One-way ANOVA-Tukey b; ^abc^ Same letters express significant difference between groups. Abbreviations: DM: diabetes mellitus RMR: resting metabolic rate, HBE: Harris-Benedict Equation, MSJ: Mifflin-St Jeor Equation, OW: Oven Equation.

Demographic data and physical examination findings are presented in Table 2.

**Table 2:** Demographic data and physical examination findings in control and diabetic groups.

Variables	Gender	Total	Control	Type 1 DM	Type 2 DM	P
Age (year)	All	41.32 ± 15.10	39.70 ± 12.99^a,b^	29.50 ± 9.33^a,c^	55.69 ± 11.74^b,c^	<0.001*
	Female	41.95 ± 13.93	38.99 ± 12.28^a,b^	30.53 ± 9.30^a,c^	54.82 ± 8.69^b,c^	<0.001*
	Male	39.77 ± 17.63	43.07 ± 15.79^a,b^	28.39 ± 9.32^a,c^	54.36 ± 17.32b^b,c^	<0.001*
Gender	All	100% (395)	44.1% (174)	26.8% (106)	29.1% (115)	
	Female	71.1% (281)	36.5% (144)	13.9% (55)	20.8% (82)	<0.001**
	Male	28.9% (114)	7.6 % (30)	12.9% (51)	8.4% (33)	
Height (cm)	All	162.08 ± 9.13	161,51 ± 8.53^a^	165.36 ± 9.26^a,c^	159.91 ± 9.15^c^	**<0.001**
	Female	158.19 ± 6.22	159.26 ± 6.53^a^	158.95 ± 5.90^b^	155.80 ± 5.19^a,b^	**<0.001**
	Male	171.66 ± 8.05	172.30 ± 8.81	172.27 ± 6.96	170.12 ± 8.90	0.432
Weight (kg)	All	73.91 ± 16.45	72.79 ± 16.35^a,b^	64.54 ± 10.82^a,c^	84.25 ± 15.17^b,c^	**<0.001**
	Female	72.55 ± 16.10	70.99 ± 16.03^a,b^	61.62 ± 1.08^a,c^	82.62 ± 13.66^b,c^	**<0.001**
	Male	77.26 ± 16.89	81.40 ± 15.33^a^	67.69 ± 10.81^a,c^	88.30 ± 18.00^c^	**<0.001**
BMI (kg/m2)	All	28.24 ± 6.36	27.93 ± 6.06^a,b^	23.60 ± 3.51^a,c^	32.98 ± 5.47^b,c^	**<0.001**
	Female	29.07 ± 6.55	28.05 ± 6.33^a,b^	24.39 ± 3.79^a,c^	34.01 ± 5.18^b,c^	**<0.001**
	Male	26.20 ± 5.35	27.38 ± 4.57^a,b^	22.76 ± 2.99^a,c^	30.44 ± 5.41^b,c^	**<0.001**
Waist circumference (cm)	All	91.75 ± 16.17	89.78 ± 14.81^a,b^	79.66 ± 9,24^a,c^	105.87 ± 12.19^b,c^	**<0.001**
	Female	92.12 ± 16.88	88.89 ± 15.26^a,b^	78.35 ± 10.03^a,c^	107.04 ± 11.59^b,c^	**<0.001**
	Male	90.84 ± 14.27	94.08 ± 11.68^a,b^	81.07 ± 8.18^a,c^	102.98 ± 13.31^b,c^	**<0.001**
SBP (mmHg)	All	125.82 ± 22.60	122.03 ± 18.43^a,b^	114.41 ± 14.72^a,c^	142.08 ± 25.32^b,c^	**<0.001**
	Female	125.95 ± 23.54	121.22 ± 18.16^a,b^	112.18 ± 15.24^a,c^	143.50 ± 26.42^b,c^	**<0.001**
	Male	125.49 ± 20.20	125.90 ± 19.52^a^	116.80 ± 13.89^b^	138.55 ± 22.33^a,b^	**<0.001**
DBP (mmHg)	All	73.82 ± 13.19	72.24 ± 14.01^a^	71.28 ± 10.93^b^	78.55 ± 12.68^a^,^b^	**<0.001**
	Female	73.73 ± 13.62	72.02 ± 14.41^a^	70.45 ± 9.94^b^	78.93 ± 13.02^a,b^	**<0.001**
	Male	74.04 ± 12.10	73.27 ± 12.07	72.18 ± 11.95	77.61 ± 11.94	0.122
PR/min	All	86.22 ± 12.93	82.41 ± 11.19^a,b^	91.92 ± 13.05^a,c^	86.72 ± 13.35^b,c^	**<0.001**
	Female	86.38 ± 12.32	83.37 ± 10.83^a^	95.16 ± 12.64^a,c^	85.77 ± 11.90^c^	**<0.001**
	Male	85.82 ± 14.37	77.83 ± 11.91^a,c^	88.41 ± 12.67^a^	89.09 ± 16.38^c^	**0.001**

* Kruskal Wallis Test-Mann Whitney U, ** Chi-square test, One-way ANOVA-Tukey b; ^abc^ Same letters express significant difference between groups. Abbreviations: DM: diabetes mellitus, BMI: body mass index, SBP: systolic blood pressure, DBP: diastolic blood pressure, PR: pulse rate, min: minute.

Laboratory findings and medications used in the control and patient groups are listed in Table 3.

**Table 3:** Laboratory findings and drugs used in control and diabetic groups.

Variables	Gender	Total	Control	Type 1 DM	Type 2 DM	P
FBG (mg/dl)	All	155,47 ± 93.93	89.61 ± 7.56^a,b^	229.83 ± 115.01^a,c^	186.59 ± 74.04^b,c^	<0.001*
	Female	146.66 ± 84.70	89.75 ± 7.56^a,b^	238.75 ± 106.64^a,c^	184.83 ± 64.26^b,c^	<0.001*
	Male	177.20 ± 110.97	88.93 ± 7.67^a,c^	220.22 ± 123.75^a^	190.97 ± 95.14^c^	<0.001*
HbA1c (%)	All	7.37 ± 2.14	5.64 ± 0.32^a,b^	9.16 ± 2.10^a,c^	8.34 ± 1.78^b,c^	**<0.001**
	Female	7.15 ± 2.07	5.64 ± 0.32^a,b^	9.17 ± 2.20^a,c^	8.45 ± 1.67^b,c^	**<0.001**
	Male	7.92 ± 2.23	5.68 ± 0.34^a,b^	9.14 ± 2.00^a,c^	8.08 ± 2.02^b,c^	**<0.001**
Blood Creatinin (mg/dl)	All	0.79 ± 0.15	0.77 ± 013^a^	0.79 ± 0.14	0.82 ± 0.17^a^	**0.009**
	Female	0.74 ± 0.13	0.73 ± 0.10^a^	0.72 ± 0.11^b^	0.78 ± 0.17^a,b^	**0.004**
	Male	0.90 ± 0.13	0.94 ± 0.12	0.88 ± 0.13	0.92 ± 0.14	0.100
^#^HDL Cholesterol (mg/dl)	All	54.29 ± 14.02	56.43 ± 15.28^a^	55.27 ± 11.38^b^	50.22 ± 13.51^a,b^	<0.001*
	Female	55.11 ± 14.47	57.93 ± 15.68^a^	55.60 ± 11.70^b^	49.90 ± 12.55^a,b^	**<0.001**
	Male	52.27 ± 12.69	49.07 ± 10.56	54.92 ± 11.12	51.00 ± 15.83	0.110
Total Cholesterol (mg/dl)	All	192.92 ± 43.35	196.88 ± 43.69^a^	178.21 ± 43.45^a,c^	200.60 ± 39.69^c^	<0.001*
	Female	193.54 ± 43.57	194.58 ± 44.03	184.02 ± 44.91	198.13 ± 41.36	0.059*
	Male	191.40 ± 42.95	208.17 ± 40.85^a^	171.94 ± 41.34^a,c^	206.73 ± 35.02^c^	<0.001*
Triglycerides (mg/dl)	All	137.28 ± 106.06	113.68 ± 76.12^a^	116.42 ± 75.78^b^	191.61 ± 142.97^a,b^	<0.001*
	Female	134.85 ± 104.59	107.66 ± 62.41^a^	116.00 ± 76.83^b^	194.57 ± 147.24a^,b^	<0.001*
	Male	143.29 ± 109.87	143.14 ± 120.33^a^	116.88 ± 75.38^a,c^	184.24 ± 133.64^c^	0.003*
LDL Cholesterol (mg/dl)	All	112.20 ± 36.59	118.71 ± 37.80^a^	99.80 ± 33.60^a,c^	113.85 ± 33.72^c^	<0.001*
	Female	112.32 ± 36.47	115.60 ± 37.07	105.52 ± 36.27	111.08 ± 35.23	0.145
	Male	111.89 ± 37.03	134.46 ± 38.22^a^	93.62 ± 29.59^a,c^	120.69 ± 32.97^c^	<0.001*
Anti-hyperlipidemic drugs	All	18.0% (71)	5.2% (9)	0.9% (1)	53.0% (61)	
	Female	19.9% (56)	4.9% (7)	1.8% (1)	58.5% (48)	<0.001**
	Male	13.2% (15)	6.7% (2)	0% (0)	39.4% (13)	
Anti-hypertensive drugs	All	29.9% (118)	12.6% (22)	10.4% (11)	73.9% (85)	
	Female	31.7% (89)	12.5(18)	10.9% (6)	79.3% (65)	<0.001**
	Male	25.4% (29)	13.3% (4)	9.8% (5)	60.6% (20)	
Insulin (unit)	All	48.6% (192)	0% (0)	100% (106)	74.8% (86)	
	Female	42.0% (118)	0% (0)	100% (55)	76.8% (63)	
	Male	64.9% (74)	0% (0)	100% (51)	69.7% (23)	
OADs	All	25.1% (99)	0% (0)	0% (0)	86.1% (99)	
	Female	24.6% (69)	0% (0)	0% (0)	84.1% (69)	
	Male	26.3% (30)	0% (0)	0% (0)	90.9% (30)	

* Kruskal Wallis Test-Mann Whitney U, ** Chi-square test, One-way ANOVA-Tukey b; ^abc^ Same letters express significant difference between group; ^#^HDL: 2 control women,1 type 2 dm women excluded. Abbreviations: DM: diabetes mellitus, FBG: fasting blood glucose, HbA1c: hemoglobin A1c, HDL: high-density lipoprotein, LDL: low-density lipoprotein, OADs: oral anti-diabetic drugs.

A comparison between the estimated and measured RMR in controls and individuals with type 1 and 2 DM is presented in Table 4.

**Table 4:** Comparison between the estimated and measured RMR in control and diabetic groups.

Variable	Gender	Type 1 DM N Female:56 Male:55			Type 2 DM N Female:91Male:45			Control N Female:149 Male:32		
		Mean	(95% CI)	p	Mean	(95% CI)	p	Mean	(95% CI)	p
Measured RMR (kcal in 24 h)	Female	1346.73	(1276.24; 1417.21)		1548.62	(1479.04; 1618.20)		1364.15	(1321.25; 1407.06)	
	Male	1597.39	(1504.81; 1689.97)		1658.97	(1503.36; 1814.58)		1572.60	(1450.95; 1694.25)	
HBE	Female	1395.66	(1366.73; 1424,59)		1477.13	(1445.67; 1508.58)		1446.31	(1420.94; 1471.68)	
	Male	1666.72	(1616.16; 1717.27)		1763.47	(1645.21; 1881.73)		1755.99	(1653.62; 1858.36)	
Difference (kcal in 24 h)	Female	48.93	(-17.20; 115.06)	0.144	−71.49	(-128.71;-14.28)	**0.015**	82.16	(51.85; 112.48)	**<0.001**
	Male	69.32	(-12.90; 151.55)	0.097	104.50	(6.02; 202.98)	**<0.038**	183.39	(112.93; 253.85)	**<0.001**
Deviation %	Female	6.83	(1.69; 11.97))	0.10	−1.24	(-5.67; 3.19)	0,580	8.92	(5.64; 12.22)	**<0.001**
	Male	7.90	(1.99; 13.81)	0.10	9.93	(2.36; 17.50)	0.12	13.29	(8.36; 18.23)	**<0.001**
MSJ	Female	1297.78	(1263.64; 1331.92)		1368.47	(1333.16; 1403.78)		1351.77	(1323.04; 1380.51)	
	Male	1618.21	(1578.18; 1658.25)		1682.94	(1590.96; 1774.92)		1683.17	(1602.54; 1763.81)	
Difference (kcal in 24 h)	Female	−48.95	(-116.97; 19.07)	0.155	−180.15	(-237.39;-122.91)	**<0.001**	−12.38	(-43.21; 18.45)	0.429
	Male	20.82	(-62.39; 104.03)	0.617	23.97	(-78.23; 126.16)	0.636	110.57	(39.69; 181.45)	**0.003**
Deviation %	Female	−0.74	(-5.66; 4.18)	0.764	−8.68	(-12.80;-4.56)	**<0.001**	1.59	(-1.55; 4.71)	0.319
	Male	5.03	(-0.89; 10.95)	0.94	5.62	(1.85; 13.09)	0.135	9.04	(4.19; 13.83)	**0.001**
OW	Female	1237.42	(1217.86; 1256.98)		1388.23	(1366.68; 1409.77)		1304.73	(1285.77; 1323.69)	
	Male	1569.40	(1538.40; 1600.40)		1779.69	(1714.60; 1844.78)		1709.28	(1650.88; 1767.68)	
Difference (kcal in 24 h)	Female	−109.31	(-175.55;-43.06)	**0.002**	−160.40	(-220.10;-100.69)	**<0.001**	−59.42	(-92.19;-26.66)	**<0.001**
	Male	−27.99	(-112.94; 56.95)	0.511	120.72	(4.54; 236.90)	**0.042**	136.68	(44.99; 228.37)	**0.005**
Deviation %	Female	−5.18	(-9.71;-0.65)	**0.026**	−6.91	(-11.17;-2.64)	**0.002**	−1.48	(-4.501.54)	0.335
	Male	2.14	(-3.94; 8.21)	0.484	12.97	(3.96; 21.98)	**0.006**	11.66	(5.20:18.13)	0.001
Ikeda Equation	Female	1274.60	(1246.99; 1302.21)		1411.77	(1380.86; 1442.68)		1342.95	(1317.40; 1368.51)	
	Male	1466.69	(1435.34; 1498.03)		1594.94	(1523.51; 1666.37)		1559.80	(1498.30; 1621.31)	
Difference (kcal in 24 h)	Female	−72.13	(-137.68;-6.57)	**0.032**	−136.85	(-193.93;-79.78)	**<0.001**	−21.20	(-51.29; 8.89)	0.166
	Male	−130.71	(-213.15;-48.26)	**0.002**	−64.03	(-174.73; 46.67)	0.247	−12.80	(-97.14; 71.54)	0.758
Deviation %	Female	−2.49	(-7.16; 2.18)	0.290	−5.66	(-9.85;-1.47)	**0.009**	1.00	(-1.97; 3.97)	0.506
	Male	−4.73	(-10.13; 0.67)	0.085	0.70	(-6.85; 8.25)	0.851	1.56	(-3.72; 6.84)	0.551

Student-t test. Abbreviations: RMR: resting metabolic rate, DM: diabetes mellitus, HBE: Harris-Benedict Equation, MSJ: Mifflin-St Jeor Equation, OW: Oven Equation.

There was no statistically significant difference between the measured RMR and HBE and in MSJ equation results in males and females with type 1 DM. Also no statistically significant differences were observed for OW equation results in males with type 1 DM.

In males with type 2DM, there was no statistical difference between the measured RMR and estimated RMR based on MSJ and Ikeda equations. However, there was a statistically significant difference in females.

There was no statistically significant difference in the MSJ and Ikeda equations in female control and Ikeda equations in males.

**Fig. 1:**
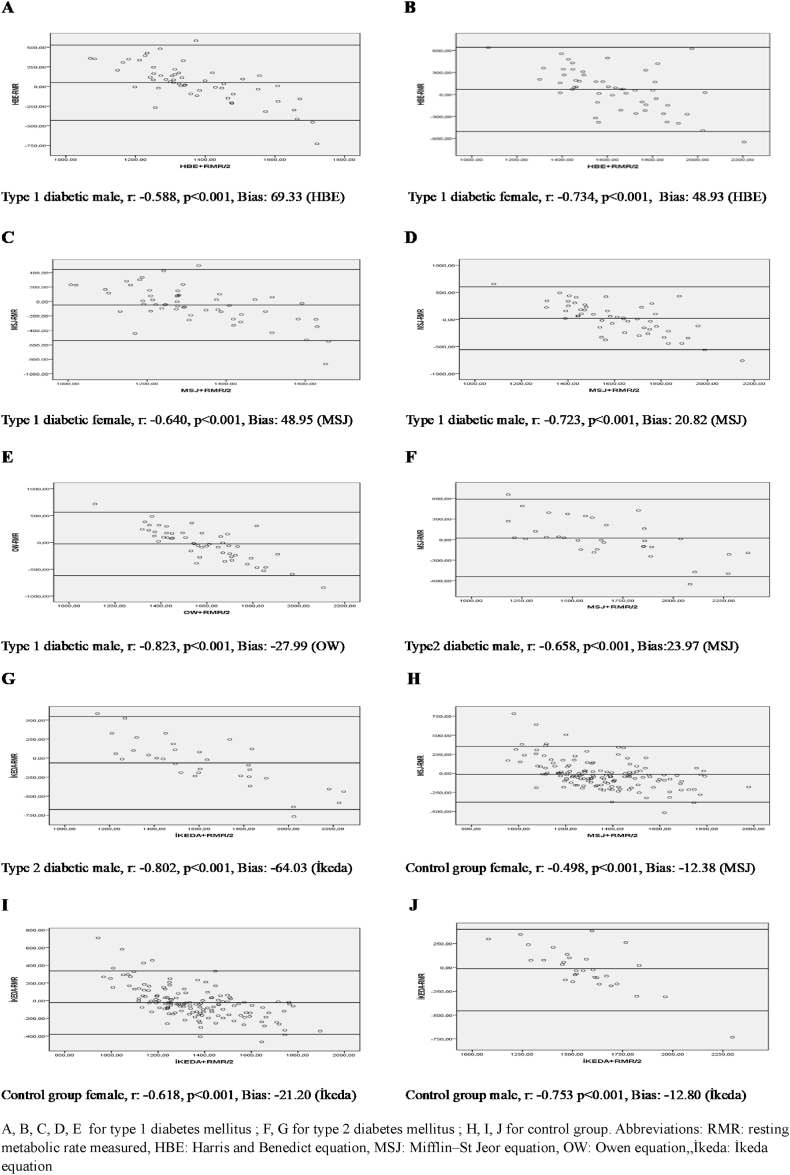
Bland Altman analysis of measured and estimated RMR values.

Fig. 1 presents the Bland–Altman analyses for groups with no significant difference between measured and estimated RMR values. The letters A, B, C, and D represent the Bland-Altman analysis for Type 1 DM; F and G are used for Type 2 DM; and H, I, and J denote the analysis in the control group. The graphs indicating levels of agreement revealed a moderate correlation between the differences and means of the estimated and measured RMR with broad limits of agreement.

The regression model developed to identify the factors that predicted RMR is presented in Table 5.

**Table 5:** Results of multiple regressions for RMR.

Model	R^2^	Adjusted R^2^	B	ε	ß	t	p	pr
Constant	0.515	*0.495*	164,100	388.535		0,422	0,673	
Type 1 DM			138,490	46.325	0,190	2990	**0,003**	0,152
Type 2 DM			95,358	40.112	0,134	2377	**0,018**	0,122
Gender (male)			94,781	39.993	0,132	2370	**0,018**	0,121
Age (year)			−7253	1.245	−0,335	−5826	**<0.001**	−0,288
Height (cm)			2626	2.200	0,074	1194	0,233	0,061
Weight (cm)			8523	1.827	0,432	4664	**<0,001**	0,234
Waist circumference (cm)			4761	2.047	0,237	2326	**0,021**	0,119
SBP (mmHg)			1524	0.887	0,106	1719	0,086	0,088
DBP (mmHg)			−0,879	1.297	−0,035	−0,678	0,498	−0,035
PR/min			−0,004	0.996	<0,001	−0,004	0,996	<0,001
FBG (mg/dl)			−0,068	0.203	−0,020	−0,333	0,739	−0,017
HbA1c (%)			−13,069	10.292	−0,086	−1270	0,205	−0,065
Blood Creatinin (mg/dl)			−67,210	98.795	−0,031	−0,680	0,497	−0,035
HDL-Cholesterol (mg/dl)			−0,020	0.949	−0,001	−0,021	0,983	−0,001
Trigliserid (mg/dl)			0,411	0.138	0,134	2983	**0,003**	0,152

Dependent variable: RMR. Abbreviations: RMR: resting metabolic rate, ε: Standard error, DM: diabetes mellitus, SBP: systolic blood pressure, DBP: diastolic blood pressure, PR: pulse rate, min: minute, FBG: fasting blood glucose, HbA1c: hemoglobin A1c HDL: high-density lipoprotein.

BMI, total cholesterol and LDL were excluded from the model due to the high variance inflation factors. Two control subjects with missing HDL-cholesterol values and one type 1 DM patient were not included in the model. Three hundred ninety-two individuals were included in the model. The analysis yielded a significant regression model (F [15, 376] 26.59, p < 0.001). The independent variables entered into the model explained 49.5% of the dependent variable.

## 4 Discussion

The research presented here is a rare study in which type 1 and 2 DM and control groups were evaluated. In this study for individuals with type 1 DM, the equation that deviated least from the measured RMR was MSJ with −0.74% in females and OW with 2.14% in males. The deviation in type 2 diabetic women was underestimated in all equations. The least deviated equations were HBE with −1.24% in females and Ikeda with 0.70% in males with type 2 DM.

In a study involving elderly individuals with type 2 diabetes, Buck et al. assessed five predictive equations, including Cunningham and HBE, and Gougeon et al. reported that RMR was underestimated and less consistent in all elderly females and with type 2 DM compared to that in males. The difference between all predicted equations and measured RMR was statistically significant [11]. Although the mean age was lower, BMI was higher in males in our study compared to that in Buck's study, and the estimated RMR was less accurate and underestimated in females with type 2 DM compared to that in males. When regulating nutritional therapy in diabetic individuals, the closest equation should be used if RMR measurement is impossible. Although the closest prediction is HBE in type 2 diabetic women, the high BMI reminded us that a different equation should be used for obese patients [12,29].

In the study reported by Steemburgo and colleagues, the average age of patients is 62, with 52.4% female. They observed a notable variation between the measured and predicted equations. Their study reported a difference of −13.6% with MSJ and −7.8% with HB. In our study, the differences with MSJ were −8.7% in females and 5.6% in males, while with HB, it was −1.2% in females and 9.9% in males. However, Steemburgo's study did not evaluate differences based on gender. Therefore, directly comparing our study with theirs would not be accurate [23].

Daly et al. reported that the HBE overestimated the basal energy expenditure measured in a cohort of 201 healthy between the ages of 18 and 67 years, males and females, by 10%–15% [16]. HB was observed to overestimate REE by 7%–24% in healthy females and by 9.2% in males of under 50, and underestimated by 0.8% males over 50 years of ages [28]. Mifflin reported that the equation that was closest to the measured RMR was OW [5].

Our study did not determine HBE to be useable in healthy subjects, and it was calculated as overestimated. The least deviated equations for the control group were Ikeda with 1.56%, MSJ with 1.59 in females, and Ikeda with 1.00% in males.We determined that the Ikeda equation was useable. For type 1 diabetic patients, the most appropriate equations were MSJ and OW. The reason that the Ikeda equation underestimates the measured RMR in individuals with type 1 DM may be due to our much higher FBG and HbA1c values.

Ferreira et al. assessed the equations of HB, FAO/WHO/UNO, MSJ, Gougeon, OW, Huang, and Rodrigues et al. in females with type 2 diabetes and observed proportional bias in all of them. However, they stated that the Owen equation yielded the result closest to the measured RMR in females with type 2 DM [17]. Although the average age and BMI were similar to ours, their study differed from our study in that it included patients with more moderate blood sugar levels and who did not use insulin. They observed that HBE was overestimated and MSJ and OW were underestimated in the equations, whereas in our study, we determined that all equations underestimated the values. This difference may be due to poor glucose control in our study.

Buscemi et al. reported that RMR was higher in patients with poorly controlled DM and that the FBG level was correlated with RMR; however, the same correlation was not observed with HbA1c. It has been stated that elevated glucose level increases glucogenesis and insulin decreases it, thus leading to decreased metabolic rate; however, the sample size was not large enough [3]. Gougen et al. determined that in obese persons with type 2 DM, FBG is an independent determinant of RMR [30]. Alawad et al. observed that RMR was higher in obese subjects with DM than it was in nonobese individuals with DM. They also reported that high HbA1c increased the RMR, but unlike Gougeon's study, they observed that FBG level was not associated with RMR [30,31]. No relationship was observed between RMR and glucose and HbA1c in obese females with type 2 DM who did not use insulin and possessed an average HbA1c of 7.7% [17]. In the study conducted between Type 2 diabetic and non-diabetic obese patients, approximately one-third of who used insulin and two-thirds who used sulfonylurea, no difference was observed in their RMR, and there was no relationship between glucose regulation and RMR. However, no evaluation was performed according to gender in the study [32]. According to the model developed in our study, FBG level and HbA1c did not predict RMR, but body weight and triglyceride levels were positively associated with RMR. Although glucose regulations were poor in our study, there was no relationship between RMR and FBG or HbA1c, and this may be due to the high rates of insulin use in our DM groups. In type 1 DM, there was no relationship between glucose regulation and HbA1c during the 3–6 month follow-up, but this led to weight gain [14]. In the study of Carnero et al. with 17 female and 16 male type 1 diabetic patients, the average age of type 1 diabetic patients was 28.6 years, HbA1c 8.2%, and the duration of diabetes and total insulin doses used were not specified. They reported that 24-h energy consumption was higher in type 1 DM than in the control group [20].The observation that RMR depends on many factors causes studies to be conducted with more specific groups. This specification, with a small research sample size, may lead to the general picture being overlooked. As we determined in our study, type 1 and type 2 DM positively predicted RMR. Long-term follow-up studies are required to examine the relationship between glucose regulation in diabetes and RMR.

In a study involving females aged 65–80 years, Sintjago et al. reported that waist circumference was a significant predictor of abdominal fat distribution and RMR and that age was negatively associated with RMR [33]. Mifflin et al. also reported that age was inversely associated with RMR [5]. In our study, as expected, waist circumference, body weight, and triglyceride levels were positive predictors of RMR, whereas age was a negative predictor.

In the study, there was a difference in RMR between the groups (as expected) with the exception of males. No significant difference was observed between the male groups. However, weight-adjusted RMR was lower in type 2 diabetics than it was in controls and type 1 DM patients. The lipogenic effect of insulin treatment and improved glycaemic control caused increased fat mass and weight gain. The mechanisms involved in this effect include the anticatabolic effect of insulin and decreased RMR when adjusted for body mass [14,22]. In our study, all participants with type 1 DM and 74.8% of the participants with type 2 DM were on insulin treatment. Additionally, the age and BMI of the type 2 DM group were higher than they were in the other groups.

## 5 Conclusions

Our results support the use of MSJ and HBE in females and MSJ and OW in males with type 1 DM.The most accurate equation for males with type 2 DM and in the group without DM appear to be the Ikeda equation. FBG level and HbA1c were not associated with measured RMR, but having type 1 or type 2 DM predicted RMR.

## Limitation

This study has many limitations. It was a single-centre study and only included a group of patients who presented to the research hospital and may not represent of the whole Turkish population. We did not consider lean body mass. Another limitation of the research is that it was conducted without considering seasonal characteristics and the uneven distribution of males and females in the study. The fact that the distribution of cases and controls is not matched demographically may affect this situation and is the limitation of this study. Many variables can affect RMR, so these findings need to be evaluated in more extensive studies.

## Source of funding

There is no funding source.

## Data availability statement

Patient data associated with the study has not been deposited into a publicly available repository. Data is available on request https://data.mendeley.com/preview/6hmxj85s5r?a=ce56e3ec-68c3-4988-89ff-f24216b4a42e.

## CRediT authorship contribution statement

**Akin Dayan:** Writing – review & editing, Writing – original draft, Project administration, Methodology, Investigation, Formal analysis, Data curation, Conceptualization. **Nilay Ergen:** Validation, Supervision, Resources, Formal analysis, Data curation. **Sami Sabri Bulgurlu:** Writing – review & editing, Resources, Investigation, Data curation.

## Declaration of competing interest

The authors declare that they have no known competing financial interests or personal relationships that could have appeared to influence the work reported in this paper.
